# Genome-Wide Analysis of *PEBP* Genes in *Dendrobium huoshanense:* Unveiling the Antagonistic Functions of *FT/TFL1* in Flowering Time

**DOI:** 10.3389/fgene.2021.687689

**Published:** 2021-07-09

**Authors:** Cheng Song, Guohui Li, Jun Dai, Hui Deng

**Affiliations:** ^1^College of Biological and Pharmaceutical Engineering, West Anhui University, Lu’an, China; ^2^Anhui Engineering Laboratory for Conservation and Sustainable Utilization of Traditional Chinese Medicine Resources, West Anhui University, Lu’an, China; ^3^College of Life Science, Anhui Agricultural University, Hefei, China

**Keywords:** *Dendrobium huoshanense*, flowering regulation, flowering locus T, homology, collinearity analysis, PEBP genes

## Abstract

Dendrobium is a semi-shade epiphytic Orchidaceae herb with important ornamental and medicinal value. Parts of the cultivation of Dendrobium germplasm resources, as well as the identification of medicinal components, are more studied, but the functional characterization of the flowering regulation in Dendrobium plants is less reported. Here, six PEBP family genes (*DhFT3, DhFT1, DhMFT, DhTFL1b, DhFT2*, and *DhTFL1a*) were identified from the *Dendrobium huoshanense* genome. The chromosome-level mapping showed that these genes were sequentially distributed on chromosomes 6, 9, 15, and 17. The paralogous gene *DhTFL1b* corresponded to *DhTFL1a*, which was determined through tandem duplication. The gene structure and conserved motif of *DhPEBP* indicated five PEBP genes apart from *DhMFT* contained four exons and three introns entirely. The phylogeny analysis showed that the PEBP gene family in *A. thaliana, O. sativa, Z. mays, S. lycopersicum*, and *P. equestris* were classified into three subclades, FT, TFL, and MFT, which maintained a high homology with *D. huoshanense*. The conserved domain of the amino acid demonstrated that two highly conserved short motifs (DPDXP and GXHR) embed in DhPEBPs, which may contribute to the conformation of the ligand binding bag. The 86th position of DhFTs was tyrosine (Y), while the 83th and 87th of DhTFL1s belonged to histidine (H), suggesting they should have distinct functions in flowering regulation. The promoter of six *DhPEBPs* contained several *cis*-elements related to hormone induction, light response, and abiotic stress, which indicated they could be regulated by the environmental stress and endogenous signaling pathways. The qRT-PCR analysis of *DhPEBPs* in short-term days induced by GA indicated the gene expressions of all *DhFTs* were gradually increased, whereas the expression of *DhTFL1* was decreased. The results implied that DhPEBPs have various regulatory functions in modulating flowering, which will provide a scientific reference for the flowering regulation of Dendrobium plants.

## Introduction

The traditional wild *Dendrobium huoshanense* experiences issues such as difficulty in natural pollination, fewer capsules, and low seed germination. Although asexual reproduction has been widely developed in the tissue culture of *D. huoshanense*, the degradation of germplasm is much more serious than sexual reproduction. Flower organs are the sexual reproduction organs of angiosperms, and the entire process of flower formation is essentially the basis for the procreation of offspring (Chen and Penfield, 2018). The flower-forming transition of plants from vegetative growth to reproductive growth is a key step to adapt to the external environment and ensure the reproduction of offspring (Liu et al., 2019). The timing of these transitions is precisely regulated by endogenous signals and the external environment, among which photoperiod signals can prompt plants to bloom at the most appropriate time (Plackett et al., 2018).

The protein encoded by phosphatidylethanolamine-binding protein (PEBP) is widely present in dicotyledonous monocots, and its family consists of three subfamilies, the FT-like, MFT-like, and TFL1-like (Auge et al., 2018). *FLOWERING LOCUS T (FT)*, as a florigen-inducing gene, has been identified in model plants such as Arabidopsis, rice, and maize (Ahluwalia and Hatsukami, 2015; Jin et al., 2019). FT is an important integration factor in the flowering regulation pathway of plants and has been one of the key genes regulating flowering (Adeyemo et al., 2019; Bi et al., 2019). It transmits signals to the downstream flower development-related *CONSTANS (CO)* by sensing the vernalization pathway, gibberellin pathway, photoperiod pathway, and autonomous regulation pathways (Melzer, 2017; Xiao et al., 2018). Under long-day conditions, *CO* induces the expression of *FT*, and the FT protein binds to *FLOWERING LOCUS D (FD)* protein in the stem end meristem (SAM) to promote the expression of *APETALA1* (*AP1*) (Li et al., 2015). *TWIN SISTER OF FT (TSF)*, the homologous gene of *FT*, regulates the early flowering of *A. thaliana* and exhibits a redundant function similar to that of overexpression of *FT* (Wang et al., 2019). Another branch of the PEBP family is the *TERMINAL FLOWER 1* (*TFL1*) subfamily, whose main function is to maintain vegetative growth and infinite growth of inflorescences. In Arabidopsis, *TFL1* controls the morphological structure of the plant by regulating the meristem genes, *LEAFY (LFY)* and *AP1*, present in SAM (Wang et al., 2017). In addition, *TFL1* plays a role in inhibiting flowering during flowering, and it exhibits completely different characteristics from FT (Jin et al., 2021). *ARABIDOPSIS THALIANA CENTRORADIALIS HOMOLOGU (ATC)* is classified into the same subclass as *TFL1* and is homologous to the snapdragon *CEN* gene. *ATC* overexpression can complement the late flowering phenotype of *tfl1*, but the *atc* mutant has no obvious early flowering phenomenon (González-Suárez et al., 2020). *Brother of FT and TFL1 (BFT)* is another member of the *TFL1* subfamily. Overexpression of *BFT* in *A. thaliana* shows delayed flowering, but the *bft* mutant does not show a similar phenotype to the *tfl1* mutant (Zhang et al., 2016). *MOTHER OF FT AND TFL1 (MFT)* is the ancestral gene of *FT* and *TFL1*. Overexpression of *AtMFT* can lead to early flowering, but it is not as significant as *FT*. In addition, MFT is specifically expressed in seeds, mainly through ABA and GA signaling pathways to participate in seed germination regulation (Yu et al., 2019). In dicotyledonous plants like *A. thaliana*, tomato, grape, and poplar, 6–9 genes are contained in this PEBP family (Luo et al., 2018). However, the number of PEBP family members in monocotyledonous plants is about three times that of dicotyledonous plants. *O. sativa* and *T. aestivum* have 17 and 30 PEBP genes, respectively. Through large fragments and genome-wide replication, plants have produced a large number of repetitive genes in the process of evolution. Some functions are redundant, some genes are silent as non-function, and some neofunctionalization genes have novel functions due to mutations (Schiessl et al., 2017). Six conserved ligand-binding sites in PEBP proteins of different species form a pocket-like structure, in which two amino acids are the key sites that determine the function of *FT/TFL1*. *TFL1* not only affects flowering time but also affects inflorescence morphology. In some plants, *TFL1* homologous genes may have different regulatory functions. For example, the DET and LF genes in pea control the flowering time and the developmental state of the apical meristem, respectively (Johnson et al., 2019).

To solve the bottleneck behind the sexual reproduction of *D. huoshanense*, the genetic background of *D. huoshanense*, the regulation mechanism of key genes related to flowering need to be clarified. Based on the previous genome sequencing, six PEBP family genes were screened from the whole genome of *D. huoshanense*. Among them, the two subfamilies FT and TFL1 have unique regulatory effects in the flowering initiation process. Phylogeny analysis showed that *MFT, FT*, and *TFL1* of *D. huoshanense* could be clustered with PEBP family of other species. By calculating the ka/ks of *DhPEBP* orthologous genes, all genes had suffered purified selections. Collinearity analysis revealed that the *FT/TFL1* of *D. huoshanense* and some PEBP family genes in *O. sativa*, *Z. mays*, and *G. max* have collinearity. Amino acid alignment analysis showed that the 86th amino acid of *DhFT2* and 87th of *DhTFL1a* were different. The promoter analysis indicated that signal elements related to stress and gibberellin binding possessed in *DhFT* and *DhTFL1*. Quantitative fluorescence analysis verified the flower-promoting effect of *DhFT1* and *DhFT3* as well as the flower-inhibiting effect of *DhTFL1a*. These studies would provide scientific reference for elucidating the different roles of PEBP family members in the flowering regulation of *D. huoshanense*.

## Materials and Methods

The tissue culture material of *D. huoshanense* used in the experiment came from the Anhui Engineering Technology Research Center of Plant Cell Engineering of West Anhui University (Lu’an City, Anhui Province). In total, 0.5 mM GA3 solution was sprayed once a day on the leaves of *D. huoshanense*. The samples were taken every 5 days, and each sample was biologically repeated three times. The control group was sprayed with clean water in the same way. After removing the leaves with scissors, we put them in liquid nitrogen and immediately froze them before transferring them to a 5mL screw tube to store them in a refrigerator at −80 degrees for later use.

### Identification of PEBP Family Genes in *D. huoshanense*

Firstly, the consensus conserved seed file (PF01161) of the hidden Markov model (HMM) was downloaded from the Pfam website^1^. Then, the HMM profile was performed as a query to identify all PBP-containing domain in *D. huoshanense* by retrieving against the genome with a threshold of e-value of < e^–3^. All candidate DhPEBPs were verified by Pfam^2^, SMART^3^, and InterPro^4^ for further confirmation. The same method was used to screen out PEBP genes in *A. thaliana, O. sativa subsp. japonica, Z. mays, S. lycopersicum*, and *P. equestris.* The genome sequence and CDS files of *A. thaliana, O. sativa subsp. japonica, Z. mays*, and *S. lycopersicum* were obtained from Ensembl Plants database^5^. The genome sequence and CDS file of *P. equestris* were got from the NCBI database^6^. Based on the sequence alignments generated by the Muscle method in MEGA software (v.6.06), all putative redundant PEBP sequences were discarded.

### The Chromosome Location and Gene Duplication Events

By using TBtools (v.1.089), the chromosome location information of the PEBP gene was obtained (Chen et al., 2020). Firstly, the gene density information from the GFF3 file was extracted, and the gene location visualization from the GTF/GFF tool was used to obtain the chromosome location map. The Genome gene dot plot tool was used to draw a dot plot of the gene duplication events. Using MEME suite^7^ for conservative motif analysis, the conservative motif of PEBP gene was obtained by searching the protein sequence. The number of motifs was set at 20, and the width of motifs was in 6 to 200, and select the default parameters to get the meme.xml file. TBtools was used to visualize the motif pattern and the gene structure analysis.

### The Phylogeny Analysis of PEBP Genes

Neighbor-joining (NJ) and maximum-likelihood (ML) methods were used to construct the phylogenetic tree of PEBP genes in *A. thaliana, O. sativa subsp. japonica, Z. mays, S. lycopersicum*, and *P. equestris*. MEGA 6 was used to align the target protein sequence (Tamura et al., 2013). The aligned file was used to construct the phylogenetic tree and bootstrap consensus tree by using the NJ method. Bootstrap replications value is set to 1,000, and the substitution model was the Poisson model. Gaps and missing dates were treated in pairwise deletion. Using IQ-TREE (v.1.6.6), a phylogenetic tree was construct based on the ML method.

### Identification of Orthologous and Paralogous Genes

Using Orthovenn2^8^, the orthologous and paralogous genes in *A. thaliana, O. sativa subsp. japonica, Z. mays, S. lycopersicum*, and *G. max* were identified and compared to obtain the phylogenetic trees to identify the PEBP homologous genes of *D. huoshanens*e. Through Orthovenn2 analysis, the common and unique clusters information of the five species is obtained. Using six DhPEBPs family genes as templates, the clusters associated with them are retrieved.

### The Pressure Analysis of Evolutionary Selection

The Ka and Ks values of the obtained orthologous gene pairs were used to calculate the ka/ks ratio of all the homologous gene pairs. Meanwhile, DnaSP (v.5.10.01) was used to recalculate each gene pair to remove the gene pair with high disproportionation value, and, finally, we obtained the homologous gene pairs with normal ka/ks ratio (Ge et al., 2017).

### The Collinearity Analysis of PEBP Genes

Based on the genome sequence and gene annotation files, we used the one-step MCScanX plug-in in TBtools to obtain the collinearity files between every two species, including collinearity, gene linkage, and basic gene replication.

### Amino Acid Alignment and the Conserved Domain Analysis

MEGA 6 was used to compare the PEBP protein sequences of *A. thaliana, O. sativa subsp. japonica, Z. mays, S. lycopersicum*, and *P. equestris*. According to the level of amino acid homology, Genedoc^9^ was used for amino acid coloring.

### The *cis*-Acting Element Analysis of *DhPEBPs*

To find out the responsive elements in the promoter, TBtools was used to extract the upstream 2,000 bp of gene sequence from the genome sequence of *D. huoshanense* (Carvalho et al., 2015). First, the GFF/GTF sequence extractor tool in the TBtools software was used to obtain the promoter regions of all genes. Then, the quick fasta extractor or filter tool was used to extract the promoter sequence of all PEBP family genes. PlantCARE^10^ was used to analyze the *cis*-regulatory elements in the sequences. The BioSequence Viewer tool was used to visualize the promoter elements of *DhPEBPs*.

### The qRT-PCR Analysis of *DhPEBPs*

RNAprep Pure Plant Kit (Takara, Japan) was used to extract total RNA from Dendrobium leaves of CK group and GA-treated groups for 5 and 10 days. An ultra-micro spectrophotometer was used to detect RNA concentration and quality. The 7,500 series real-time fluorescent quantitative PCR (Bio-RAD, America) was used for the quantitative analysis. The CDS sequences of the six genes *DhFT3, DhFT1, DhMFT, DhTFL1b, DhFT2*, and *DhTFL1a* were obtained from the genome of *D. huoshanense*, and the primers for the fluorescent qPCR were designed respectively (Supplementary Table 1). The total RNA of the above samples was reverse transcribed into cDNA with PrimeScript RT reagent Kit (Takara, Japan). A total of 20 μl reaction system was used for qPCR: 10 μl SYBR Premix Ex Taq II (2×), 2 μl cDNA, 0.8 μl DhFT-RT-F, and DhFT-RT-R. The PCR reaction program was as follows: 50°C 2 min, 95°C 30 s, 95°C 5 s, 60°C 34 s, 40 cycles; and 72°C 10 min. With β-actin as the internal reference gene, the 2-^ΔΔCt^ method was used to calculate the relative gene expression, and the experiment was repeated three times.

## Results and Discussion

### Identification of PEBP Family in *D. huoshanense*

Many studies had confirmed the PEBP genes were involved in flowering regulation (Ahluwalia and Hatsukami, 2015). We used the established hidden Markov model of PEBP protein (PF01161) to conduct preliminary screening of PEBP genes in the whole genome of *D. huoshanense* (Han et al., 2020). A total of six PEBP family genes were identified, and we further verified the specific PBP-containing domain through pfam, SMART, and the InterPro database, which confirmed these six genes were PEBP genes.

### Gene Duplication of *D. huoshanense* and the Chromosomal Location of the PEBP Genes

**FIGURE 1:**
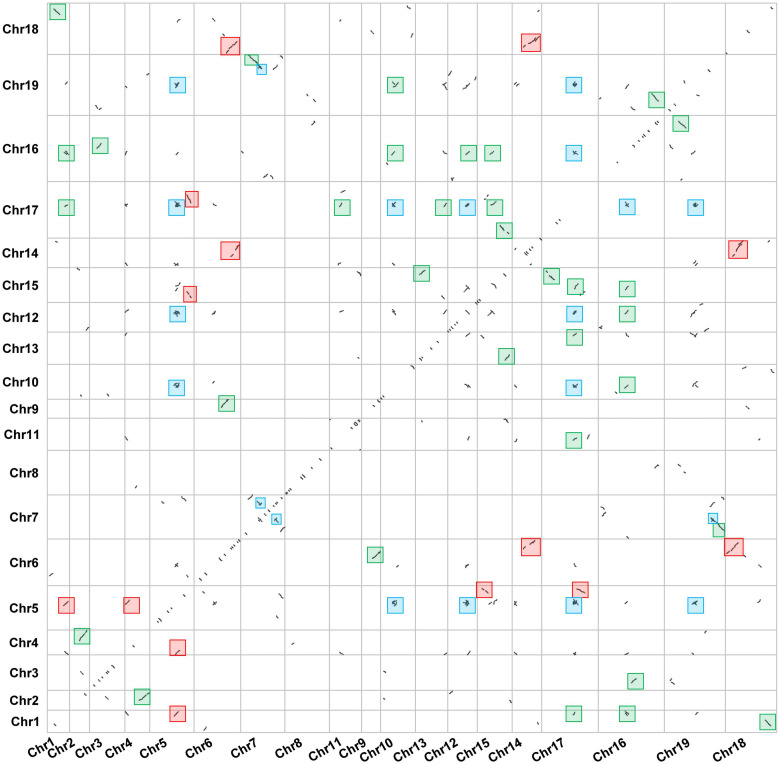
The genome dotplot of *D. huoshanense.* The dot lines denote the duplication events that occur between two chromosomes. The blue boxes represented the ancient WGD events. The red boxes represented the large-scale WGT events. The green boxes represented the recent WGD events.

**FIGURE 2:**
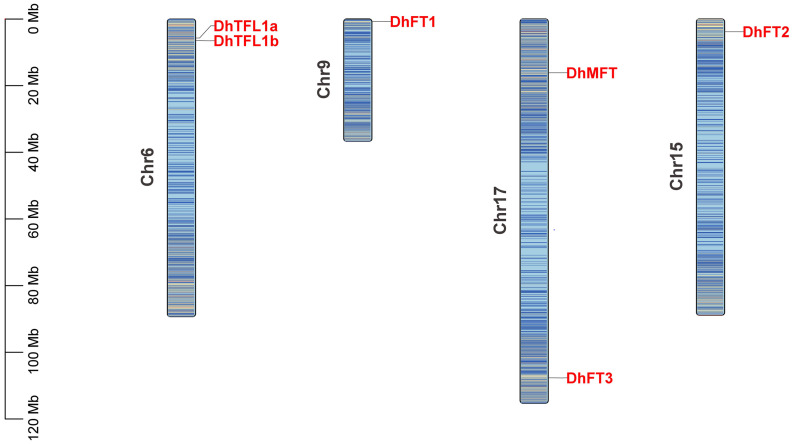
The chromosome location of PEBP genes of *D. huoshanense.*

Large-scale gene duplication and recombination are one of the important driving forces of species evolution (Jiang et al., 2020). However, few reports have focused on the gene duplication event in Dendrobium plants. In this study, we analyzed the gene duplication events in the evolution of *D. huoshanense*. The dot plot shows that *D. huoshanense* has experienced at least two large-scale WGD events since ancient times. There are a large number of WGD events that occurred in chromosome 1, chromosome 4, chromosome 16, chromosome 17, and chromosome 19. A large number of WGT events in chromosome 5, chromosome 6, chromosome 7, chromosome 14, and chromosome 18 were observed. In addition, the ancient γ-WGD events also appeared in great numbers, including chromosome 5, chromosome 10, chromosome 12, chromosome 19, and chromosome 17. The tandem duplication were scattered on the diagonal (Figure 1). These results indicated that *D. huoshanense* has undergone such ancient polyploidization events to adapt to changes in the external environment, which are consistent with previous results (Ospina-Zapata et al., 2020). The phylogeny tree indicated that two ancient PEBP duplication events in the lineage leading to the common ancestor of angiosperms after its split with gymnosperms. The first duplication gave rise to the MFT-like subfamily and the ancient lineage of the TFL1-like and FT-like subfamilies, which experienced a second duplication to create the two subfamilies. The *TFL1* ancestor underwent two separate duplication events in the common ancestor of angiosperms, which created three daughter lineages corresponding to *BFT*, *TFL1*, and *ATC* in *A. thaliana* (Wang et al., 2015). The chromosome mapping showed that the PEBP family genes were distributed on chromosomes 6, 9, 17, and 15, among which *DhFT1, DhFT2*, and *DhFT3* were located on chromosomes 9, 15, and 17, respectively. The two tandem duplicated genes of the DhTFL1 family are located on chromosome 6 (Figure 2).

### Gene Structure Analysis and Conserved Motifs of *DhPEBPs*

**FIGURE 3:**
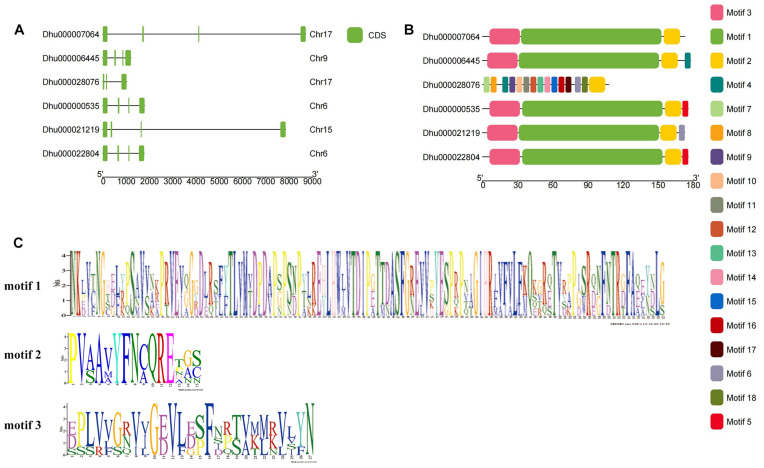
The gene structure and the conserved motif of PEBP genes in *D. huoshanense.* **(A)** The gene structure of exon and intron. **(B)** The conserved motif distributed in PEBP genes. **(C)** The composition of three main motifs.

The gene structure of *DhPEBP* showed that, except for *DhMFT*, the other five genes contained four exons and three introns (Figure 3A). The conserved motifs in the six DhPEBP proteins were identified using the MEME suite (Figure 3B). In total, 20 motifs were identified in DhPEBP proteins, named motifs 1 to 20, and the motifs identified were 6 to 121 amino acids in length. Motif 1, motif 2, and motif 3 are the three main conserved domains in the PEBP gene (Figure 3C). The number of mainly conserved motifs in each PEBP varied between three and four, indicating that the same subgroup of PEBP protein members shared one or more identical motifs. All of the PEBP genes contained motif 2. Except for the *DhMFT*, the other five PEBP genes had similar motif composition, which suggested their similar functions. However, some motifs were only presented in *DhMFT*, indicating that they may perform its particular functions (Zhao et al., 2020).

### The Phylogenetic Analysis of PEBP Genes

**FIGURE 4:**
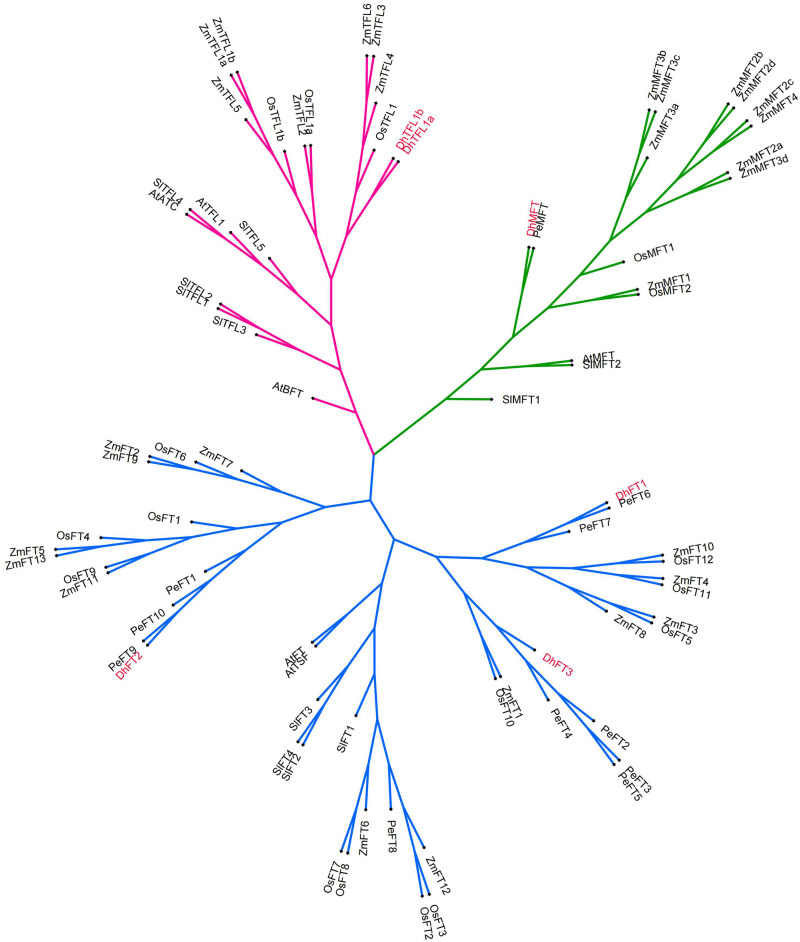
The phylogenic tree of five species by ML method.

In this experiment, MEGA 6 was used to perform homology alignment and phylogeny analysis of 81 PEBP-like protein sequences from six species. The evolutionary tree constructed by the NJ method showed that these genes were clearly divided into three clades. According to the classification of the PEBP family in *A. thaliana*, we divided these genes into FT subclade, TFL1 subclade and MFT subclade (Supplementary Figure 1). *D. huoshanense* PEBP protein maintained high homology with genes in the same clade of other species. To improve the reliability and accuracy of the established phylogenetic tree, we used the IQ-TREE tools to build an ML tree based on the optimal model (Nguyen et al., 2015). The optimal model is determined by calculation to be JTT + G4. Using this mode, further phylogeny analysis showed that *DhTFL1* is the closest to *OsTFL1*, *DhMFT* is the closest to *PeMFT*, and *DhFT1*, *DhFT2*, and *DhFT3* are the closest to *PeFT6*, *PeFT9*, and *PeFT4*, respectively. These results are also consistent with the adjacent evolutionary relationship between Dendrobium and Phalaenopsis genus (Figure 4).

### Identification of Homologous Genes With Selection Pressure Analysis

To further clarify the homologous duplication of *DhPEBPs* in the evolutionary process, the plausible functions of homologous genes were speculated. The Orthovenn 2 was used to analyze the homology of five species of *A. thaliana, D. huoshanense, Z. mays, S. lycopersicum*, and *G. max*. The results showed that these species formed 23,615 clusters, including 23,049 orthologous clusters (at least contains two species) and 566 single-copy gene clusters. Among them, the genes from *D. huoshanense* were distributed in 8,714 clusters (Supplementary Figure 2). We further analyzed the clusters related to *DhPEBP* and found that *DhPEBPs* scattered in cluster 734, cluster 2,452, and cluster 19,662, indicating these genes are orthologous genes (Supplementary Figure 3). In order to further reveal the evolutionary selection pressure of these homologous genes, we used DnaSP 5.0 to calculate the ka and ks values of each gene pair and found that the ka/ks ratio of all gene pairs was far less than 1, indicating that they were all subjected to a strongly purified selection (Table 1).

**TABLE 1:** The orthologous and paralogous genes with Ka/Ks ratio among five species.

Seq_1	Seq_2	Ka	Ks	Ka/Ks
Dendrobium_huoshanense| Dhu000000535	Zea_mays| Zm00001d050649	0.1259	2.8550	0.0441
Dendrobium_huoshanense| Dhu000000535	Zea_mays| Zm00001d052537	0.1302	3.1977	0.0407
Dendrobium_huoshanense| Dhu000000535	Glycine_max| GLYMA_03G194700	0.1653	2.1969	0.0753
Dendrobium_huoshanense| Dhu000000535	Glycine_max| GLYMA_10G071400	0.1927	6.3124	0.0305
Dendrobium_huoshanense| Dhu000000535	Glycine_max| GLYMA_11G209500	0.2028	2.7745	0.0731
Dendrobium_huoshanense| Dhu000000535	Arabidopsis_thaliana| AT5G03840	0.2000	3.0282	0.0661
Dendrobium_huoshanense| Dhu000000535	Glycine_max| GLYMA_19G194300	0.1676	2.2117	0.0758
Dendrobium_huoshanense| Dhu000022804	Zea_mays| Zm00001d052537	0.1303	3.1638	0.0412
Dendrobium_huoshanense| Dhu000022804	Zea_mays| Zm00001d050649	0.1260	2.5464	0.0495
Dendrobium_huoshanense| Dhu000022804	Glycine_max| GLYMA_11G209500	0.2028	2.2936	0.0884
Dendrobium_huoshanense| Dhu000022804	Glycine_max| GLYMA_10G071400	0.1927	2.8590	0.0674
Dendrobium_huoshanense| Dhu000022804	Glycine_max| GLYMA_03G194700	0.1654	1.9347	0.0855
Dendrobium_huoshanense| Dhu000022804	Arabidopsis_thaliana| AT5G03840	0.2001	2.6514	0.0755
Dendrobium_huoshanense| Dhu000022804	Glycine_max| GLYMA_19G194300	0.1677	1.9457	0.0862
Dendrobium_huoshanense| Dhu000007064	Zea_mays| Zm00001d006116	0.1734	1.2652	0.1371
Dendrobium_huoshanense| Dhu000006445	Zea_mays| Zm00001d017134	0.1505	1.8798	0.0800
Dendrobium_huoshanense| Dhu000006445	Glycine_max| GLYMA_19G108100	0.2645	3.7647	0.0703
Dendrobium_huoshanense| Dhu000006445	Arabidopsis_thaliana| AT4G20370	0.2314	3.5402	0.0654
Dendrobium_huoshanense| Dhu000006445	Arabidopsis_thaliana| AT1G65480	0.2392	3.0163	0.0793
Dendrobium_huoshanense| Dhu000006445	Solanum_lycopersicum| Solyc03g063100.1	0.2407	2.8471	0.0845
Dendrobium_huoshanense| Dhu000000535	Dendrobium_huoshanense| Dhu000022804	0	0.0236	0

### Colinearity and Microsynteny Analysis

**FIGURE 5:**
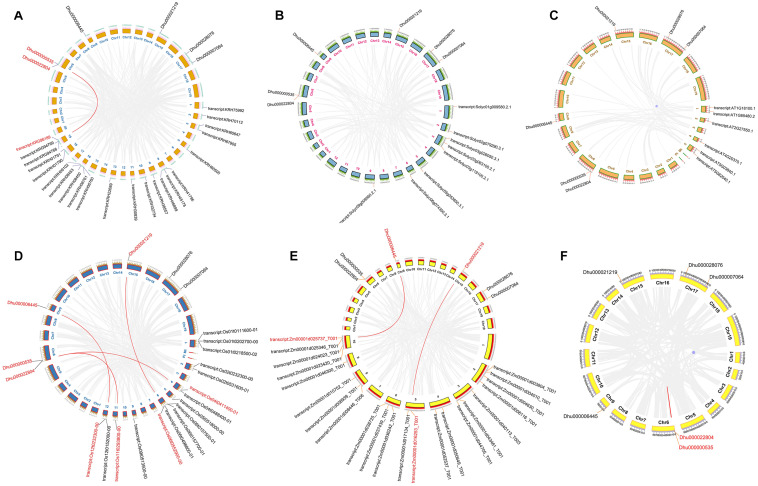
The collinearity and mcirosynteny of *D. huoshanense* and other species. **(A)** *D. huoshanense* and *G. max.* **(B)** *D. huoshanense* and *A. thaliana.* **(C)** *D. huoshanense* and *Z.mays.* **(D)** *D. huoshanense* and *S. lycopersicum.* **(E)** *D. huoshanense* and *O. sativa.* **(F)** *D. huoshanense* and *D. huoshanense.*

Collinearity analysis is often used to reveal the genetic relationship between the same species or several different species. In this experiment, we performed microsynteny analysis on the genomes of *A. thaliana, D. huoshanense, Z. mays, S. lycopersicum*, and *G. max.* It was indicated that *DhTFL1/TFL2* and *GmTFL1* (KRG96189) have collinearity, *DhFT2* and *OsFT8*, *DhFT1*, and *OsFT2*, and *DhTFL1/TFL2* and *OsTFL1b* have collinearity. *DhFT1* and *ZmFT3*, *DhFT2*, and *ZmFT13* have collinearity (Figure 5). In addition, a pair of tandem duplicated genes *DhTFL1* and *DhTFL2* are paralogs. These results indicated there was a homologous evolutionary relationship on PEBP genes between *D. huoshanense* and other species.

### Amino Acid Alignment and the Conserved Domain Analysis

**FIGURE 6:**
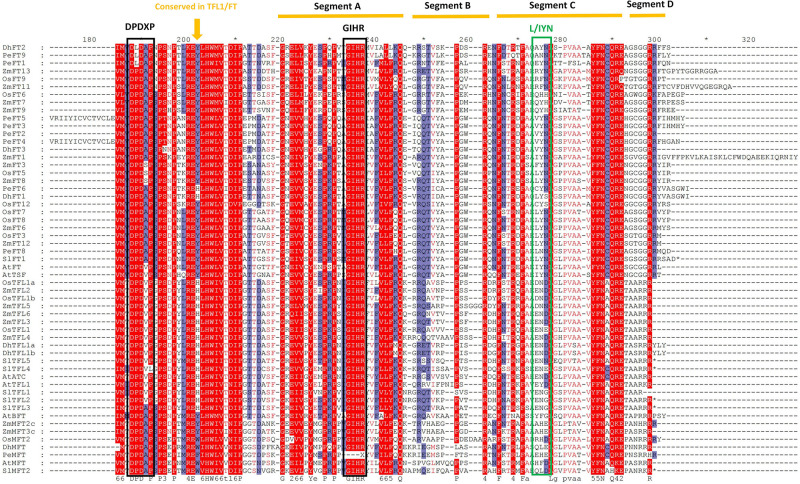
The amino acid comparison and conserved domains of PEBP proteins. The brown arrow indicates a key amino acid residue that determines FT-like and TFL1-like functions. Black boxes denote the conserved DPDxP, GIHR motif, and L/IYN motif. Underlines represent segments A–D.

Within amino acid comparison, the conserved structural domains of some proteins could be obtained, accompanied by the conserved catalytic sites and DNA-binding sites for the subsequent research (Li et al., 2014). In this experiment, we performed the amino acid alignment on the aligned sequences of 81 proteins from six species. It was indicated that the 86th position of DhFTs was tyrosine (Y), while 83th and 87th positions of of *DhTFL1s* were both histidine (H), suggesting they should have distinct functions in flowering regulation (Figure 6). PEBP proteins are characterized by the presence of two highly conserved short motifs, DPDxP and GxHR, which presumably contribute to the conformation of the ligand binding pocket (Guo et al., 2015; Mackenzie et al., 2019). It was reported that substitution of the single amino acid, Tyr85 to His, in FT partially converts FT function to TFL1 function probably through discrimination of structurally related interactors (Yang et al., 2019). In addition, the amino acid sequence encoded by the fourth exon plays a critical role to determine FT/TFL1 protein functions, which are divided into four segments (A–D). Segment B and segment C containing the LYN/IYN triplet conserved motif are especially important for the determination of functional specifcity between FT and TFL1.

### Analysis of *cis*-Acting Elements

**FIGURE 7:**
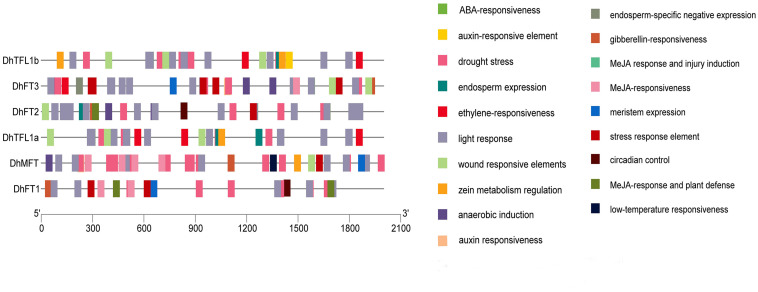
The *cis*-acting element of *DhPEBPs.*

The PLANTCARE service was performed to analyze the upstream promoter sequences of six *DhPEBP* genes to discover hormone-responsive elements related to flowering regulation, such as GARE elements (Figure 7). The results showed that the promoter region of *DhFT1, DhFT2*, and *DhMFT* all contained a GARE-motif (TCTGTTG), while that of *DhFT1* and *DhFT3* both contained a P-box motif (CCTTTTG), implying that they may be involved in flowering regulation under the GA signaling pathway (Supplementary Table 1).

### The Expression Profile of *DhPEBPs* Induced by GA

**FIGURE 8:**
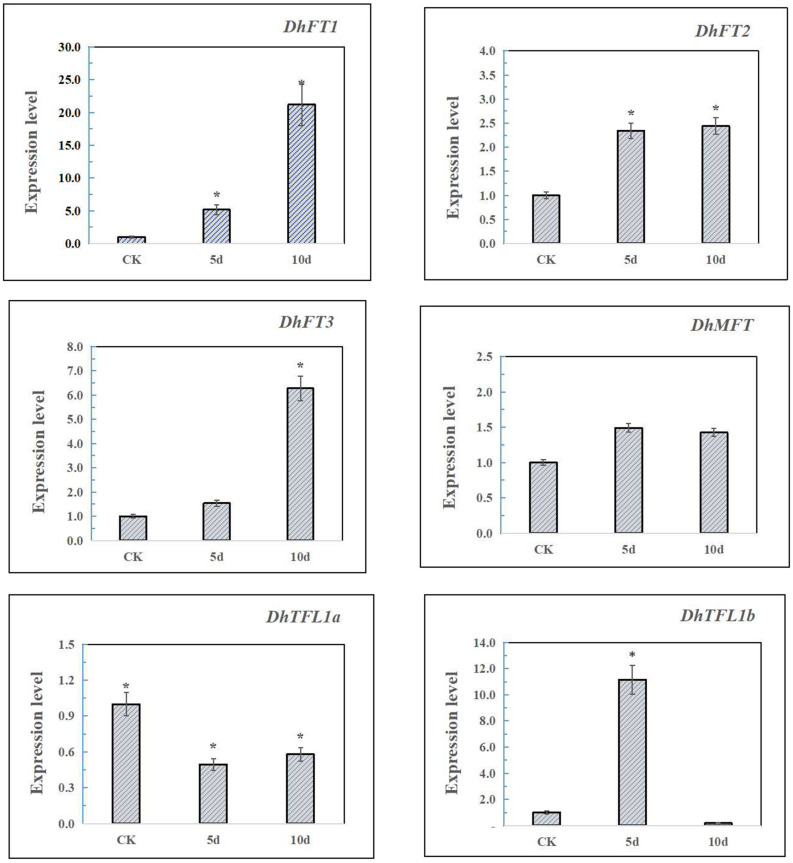
The qRT-PCR analysis of *DhPEBPs*. Asterisks mean that the expression levels have significant differences compared with CK.

In the experiment, qRT-PCR was performed on Dendrobium leaves treated with gibberellin for 5 and 10 days and the CK group (Figure 8). The results showed that *DhFT1* and *DhFT3* were strongly induced by GA treatment at 10 days, and the expression levels were increased by 21.2 times and 6.2 times, respectively. The expression level of *DhFT2* remained stable after 5 days of treatment. The expression of *DhMFT* was relatively stable, with a slight increase at 5 days. Due to the inhibition of negative feedback regulation, the expression of *DhTFL1* decreased rapidly after GA treatment, especially *DhTFL1b* was hardly expressed at 10 days. The results indicated that *DhFT* and *DhTFL1* may have different regulatory roles in the flowering regulation of *D. huoshanense*.

## Conclusion

Six PEBP genes were isolated from the *D. huoshanense* genome. The chromosome mapping suggested that the genes were distributed on chromosomes 6, 9, 15, and 17. The paralogs *DhTFL1b* and *DhTFL1a* that obtained by tandem duplication exhibited similar function. Gene structure and the conserved motif analysis further indicated 5 PEBP genes apart from *DhMFT* contained four exons and three introns entirely. The phylogeny showed that PEBP genes in *A. thaliana*, *Z. mays*, *S. lycopersicum*, *O. sativa*, and *D. huoshanense* can be classified into three subclades, FT, TFL, and MFT, which maintained a high homology with the same family in other species. The conserved domain of the amino acid demonstrated that two highly conserved short motifs DPDXP and GXHR embed in DhPEBPs. The 86th position of DhFTs was tyrosine (Y), while the 83th and 87th positions of DhTFL1s were both histidine (H), suggesting they had distinct functions in flowering regulation. The promoter analysis of six DhPEBPs revealed that several cis-elements related to hormone induction, light response and abiotic stress may be involved in the regulation of environmental stress and endogenous signaling pathways. The RT-PCR analysis in short-term days treated with GA3 indicated that the gene expressions of all *DhFTs* were gradually increased, whereas *DhTFL1* were decreased. Taken together, *DhPEBPs* have various regulatory functions in modulating flowering.

## Data Availability Statement

The datasets presented in this study can be found in online repositories. The names of the repository/repositories and accession number(s) can be found in the article/Supplementary Material.

## Author Contributions

CS and HD designed the research. CS, GL, and JD conducted the experiments. CS and GL analyzed the data. CS wrote the manuscript. HD and CS revised the manuscript, improved the English, and acquired the funding. All authors have read, reviewed, and approved the submitted version.

## Conflict of Interest

The authors declare that the research was conducted in the absence of any commercial or financial relationships that could be construed as a potential conflict of interest.

## Footnotes

1 http://pfam.sanger.ac.uk/family/PF01161

2 http://pfam.xfam.org/search#tabview=tab1

3 http://smart.embl-heidelberg.de/

4 http://www.ebi.ac.uk/interpro/search/sequence/

5 http://plants.ensembl.org/index.html

6 https://www.ncbi.nlm.nih.gov/genome?LinkName=bioproject_genome&from_uid~$=$~192198

7 https://meme-suite.org/

8 https://orthovenn2.bioinfotoolkits.net/

9 https://www.psc.edu/biomed/genedoc

10 http://bioinformatics.psb.ugent.be/webtools/plantcare/html/

## Supplementary Material

Supplementary Figure 1 The phylogenic tree of five species by NJ method.

Supplementary Figure 2 The orthologous clusters of five species and the main clusters involved in DhPEBPs.

Supplementary Figure 3 Orthologous genes related to DhPEBPs in several clusters.
