# Supplementary figures and images for: Genome-Wide Analysis of PEBP Genes in Dendrobium huoshanense: Unveiling the Antagonistic Functions of FT/TFL1 in Flowering Time
Source: Front Genet. 2021 Jul 9;12:687689. doi: 10.3389/fgene.2021.687689 (PMC8299281; doi:10.3389/fgene.2021.687689)

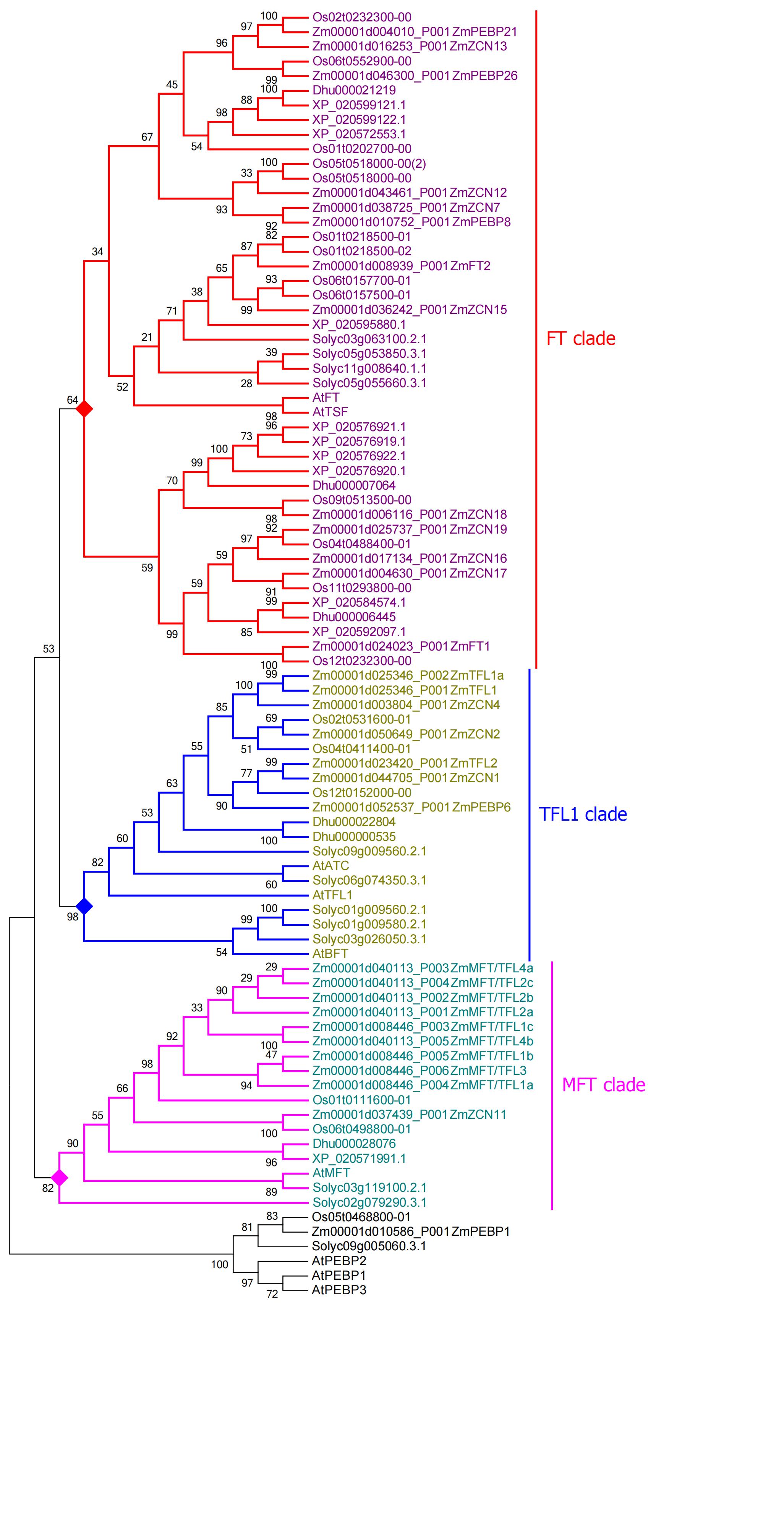

Supplement: Supplementary Figure 1 — The phylogenic tree of five species by NJ method. [file Image_1.JPEG]

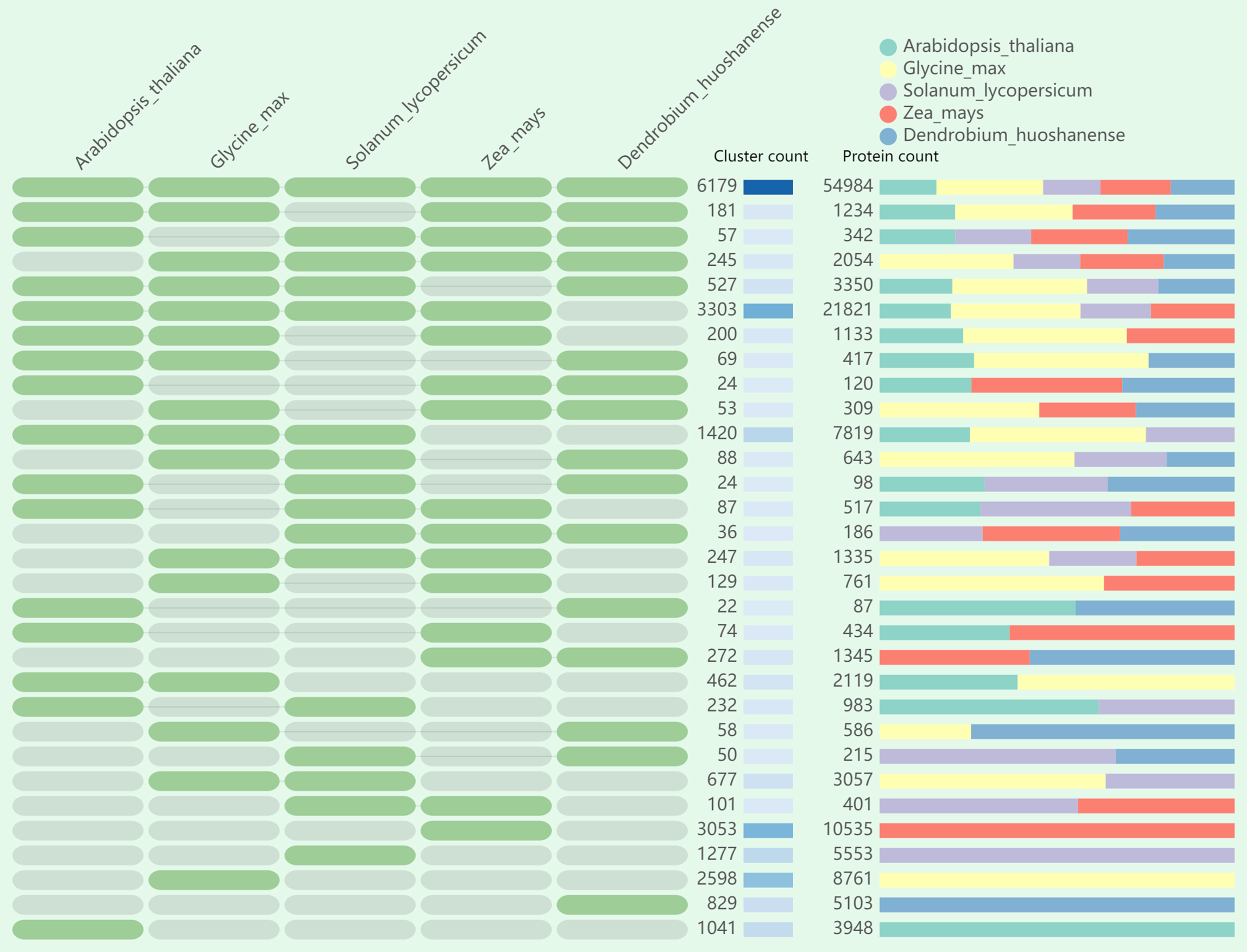

Supplement: Supplementary Figure 2 — The orthologous clusters of five species and the main clusters involved in DhPEBPs. [file Image_2.JPEG]

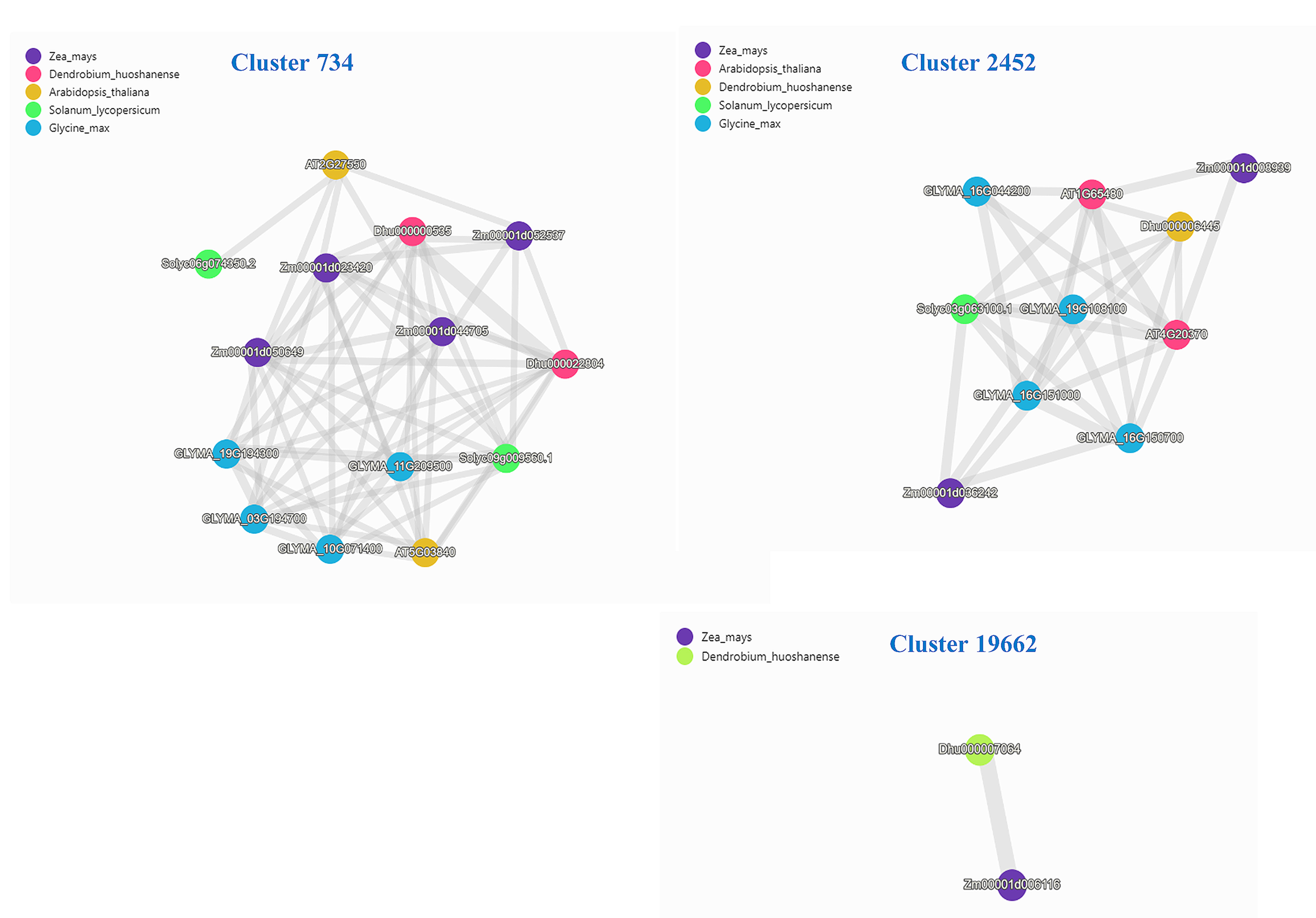

Supplement: Supplementary Figure 3 — Orthologous genes related to DhPEBPs in several clusters. [file Image_3.JPEG]
